# Sex hormones and number processing. Progesterone and testosterone relate to hemispheric asymmetries during number comparison

**DOI:** 10.1016/j.yhbeh.2019.07.001

**Published:** 2019-07-20

**Authors:** Belinda Pletzer, Seiina Jäger, Stefan Hawelka

**Affiliations:** Department of Psychology & Centre for Cognitive Neuroscience, University of Salzburg, Salzburg Austria

**Keywords:** Number magnitude processing, Unit-decade compatibility effect, Global-local processing, Sex differences, Sex hormones, Hemispheric asymmetries

## Abstract

Like many visual stimuli, multi-digit numbers are of a hierarchical nature, with whole number magnitudes depending on individual digit magnitudes. Accordingly, multi-digit numbers can be processed in a holistic (whole number magnitudes) or decomposed manner (digit magnitudes). The compatibility effect during number comparison serves as an indicator of decomposed processing. It is characterized by impaired performance for items where the larger number contains the smaller unit-digit. We were recently able to demonstrate, that the compatibility effect indeed depends on an individual’s tendency to process visual hierarchical stimuli on a global or local level. Accordingly, factors affecting global-local processing, should also affect number magnitude processing, i.e. the compatibility effect. Among these factors are hemispheric asymmetries, sex differences and sex hormones (estradiol, progesterone, testosterone). In the present study 39 men and 37 naturally cycling women in their luteal cycle phase completed a number comparison task with stimuli randomly presented to the left and right hemifield. As in previous studies, we observed a larger compatibility effect in the right hemifield (left hemisphere) than in the left hemifield (right hemisphere) and in men than in women. However, this is the first study to evaluate die effects of sex hormones on hemispheric asymmetries during number comparison. We found progesterone to relate to increased hemispheric asymmetries in men, but decreased hemispheric asymmetries in women. Additionally, testosterone was negatively related to hemispheric asymmetries in women’s compatibility effect in reaction times. These results add to the growing evidence that sex hormones relate to hemispheric asymmetries in cognitive functions.

## 1 Background

Sex hormone receptors are distributed Üiroughout the brain and especially concentrated in key areas for cognition, like the hippocampus and prefrontal cortex (Barth et al., 2015). Thus, sex hormone influences on cognitive functions have attracted considerable research interest (see Boss et al., 2014 for a review). Testosterone has been related to visuo-spatial processing, although the specific nature of the relationship is still a matter of debate (Levine et al., 2016). Estradiol on the other hand has been implicated in memory function (Luine, 2014). However, sex hormone influences on number processing have received little attention so far. The current study focuses on the role of testosterone, estradiol and progesterone (in short: sex hormones) in number magnitude processing.

In Arabic numerals, multi-digit numbers consist of individual digits, e.g. a two-digit number consists of a decade digit and a unit digit. Traditionally, it has been assumed, that the processing of multi-digit number magnitudes happens in a holistic way, i.e. their magnitude is processed as a whole on a logaridimic mental number line (Brysbaert, 1995; Dehaene et al., 1990) rather dian separately by digit. A task commonly used to study the processing of number magnitudes is the number comparison task in which participants have to identify the larger of two numbers. One effect in number comparison consistent with the holistic model is the distance effect. Performance in identifying the larger number is the worse, the smaller the numerical distance between the two numbers. The numerical distance effect occurs during both single-digit and multi-digit number comparison (Dehaene, 1989; Nuerk et al., 2001; Reynvoet and Brysbaert, 1999).

During multi-digit number comparison, however, a second effect challenges the universality of the holistic model of number magnitude processing, i.e. the **unit-decade compatibility effect** (Nuerk et al., 2001). The unit-decade compatibility effect describes the fact that comparing two-digit numbers is easier, i.e. performance is better, when the larger number contains the larger unit digit (compatible item, e.g. 78 vs. 21) than when the larger number contains the smaller unit digit (incompatible item, e.g. 71 vs. 28). If numbers were processed in a holistic fashion, interference effects of single digit magnitudes on performance should not emerge. Accordingly, the compatibility effect supports the idea that unit and decade digit magnitudes are processed separately, i.e. a decomposed model of number magnitude processing seems more plausible.

The hybrid model suggests that decomposed and holistic processing occur in parallel and the extent to which one process exceeds over the other is determined by the type of task (Verguts and De Moor, 2005; Moeller et al., 2011). For instance, it has been demonstrated that the compatibility effect varies depending on stimulus characteristics. If the two numbers to compare are spaced farther apart, the compatibility effect decreases (Pletzer et al., 2016, Pletzer et al., 2019a). Accordingly, the compatibility effect can be used as a measure for the amount of decomposed processing present in a certain situation (Moeller et al., 2011). It has also been suggested that individual characteristics determine the tendency towards holistic vs. decomposed processing (e.g. Huber et al., 2017).

The possibility of holistic and decomposed processing occurring in parallel reveals similarities to other visual processing tasks. Like multi-digit numbers, most stimuli in everyday life are hierarchical, with a global pattern emerging from local stimulus parts. Research on the parallel processing of global vs. local stimulus aspects has a long tradition and mechanisms discovered to influence global vs. local visual processing may also affect the processing of multi-digit numbers. Global-local processing is commonly studied using visual hierarchical stimuli, in which a large global shape is made up of small local shapes (e.g. a triangle made out of circles; Navon, 1977). Navon discovered that such visual hierarchical stimuli are predominantly processed on a global level, since targets at the global level are discovered faster than targets at the local level (**global advantage effect**).

Like Navon stimuli, numbers can be viewed as visual hierarchical stimuli, as the whole number magnitude is determined by the place and value of the individual digits. Indeed it has been demonstrated, that the unit-decade compatibility effect as a measure of decomposed number processing is negatively related to the global advantage effect, especially in women (Pletzer et al., n.d.-b). Women with a stronger tendency towards local visual processing also show more decomposed number processing, while women with a stronger tendency towards global visual processing show more holistic number processing. If the tendency towards holistic vs. decomposed number processing and the tendency towards global vs. local visual processing are indeed related, two new research directions regarding number processing emerge.

First, factors affecting the global advantage effect, should also affect the unit-decade compatibility effect. The global advantage effect is modulated by several factors including stimulus characteristics, such as the hemifield stimuli are presented in and individual characteristics, such as the sex or hormonal status of the participants. Second, these visual attentional processes during number processing and their influencing factors can be mapped using eye-tracking techniques. We followed up on both of these directions in a study consisting of two parts/tasks - one optimized for assessing hemispheric asymmetries during number processing, the second optimized for tracking eye-movements during number processing. In the current manuscript we describe the first part of this study, while the second part is described in Schulte et al. (n.d.). Accordingly, the current manuscript deals with the question, whether factors influencing the global advantage effect, i.e. hemispheric asymmetries, sex and sex hormones, also affect the unit-decade compatibility effect.

A functional hemispheric asymmetry, occurs when one hemisphere is more specialized for a certain cognitive function than the other. For instance, the left hemisphere is more specialized for verbal functions, while the right hemisphere is more specialized for visuo-spatial functions (see Ocklenburg and Gunturkun, 2012 for a review). Hemispheric asymmetries can be observed using neuroimaging techniques (EEG, fMRI, PET), when brain activation during a cognitive task is stronger in one hemisphere than the other (lateralization). Behaviorally, hemispheric asymmetries are studied by means of visual hemifield experiments, in which half of the stimuli are presented to the left hemifield, the other half of the right hemifield. Due to the crossing of fibres in the chiasma opticum, information presented to the left hemifield is processed in the right hemisphere, while information presented to the right hemifield is processed in the left hemisphere. A functional hemispheric asymmetry occurs, if task performance is better for stimuli presented to one hemifield compared to the other.

Hemispheric asymmetries in global-local processing are especially well established. In multiple behavioral visual hemifield, EEG, PET and fMRI experiments, it has been demonstrated that the right hemisphere shows a specialization for global processing, while the left hemisphere is more specialized on local processing (Fink et al., 1996; Robertson and Lamb, 1991). Accordingly, the global advantage effect is larger when stimuli are processed in the right hemisphere, compared to processing in the left hemisphere (Han et al., 2002; Robertson and Lamb, 1991; see van Kleeck, 1989 for a meta-analysis). If local visual processing leads to more decomposed number processing, one would expect a stronger unit-decade compatibility effect, when processing occurs in the left hemisphere. As yet, only one study (Harris et al., 2018) investigated the influence of hemifield presentation on the compatibility effect. Indeed, they found the compatibility effect to be stronger, when items were presented to the right hemifield compared to the left hemifield. However, one limitation of this study is that it lacked a control for eye fixations, which would ascertain processing in the respective hemispheres.

Hemispheric asymmetries in the global advantage effect may be closely related to effects of gender on global-local processing. Some studies proposed, that women show an advantage in cognitive functions lateralized to the left hemisphere (e.g. verbal abilities), while men show an advantage for cognitive functions lateralized to the right hemisphere (e.g. spatial abilities) (Moffat and Hampson, 1996; Clemens et al., 2006; Tomasi and Volkow, 2011; see Andreano and Cahill, 2009 for a review). Accordingly, it has been hypothesized that women show an advantage in the processing of local stimulus aspects, while men show an advantage in the processing of global stimulus aspects. Indeed several studies demonstrate a stronger global advantage effect in men than in women (Basso and Lowery, 2004; Pletzer et al., 2014; Razumnikova and Vol’f, 2011; Roalf et al., 2006; Scheuringer and Pletzer, 2016), but non-significant results have also been reported (Kimchi et al., 2009; Pletzer et al., 2017).

One possible explanation for the inconsistencies between studies is the fact that hormonal status of female participants was hardly controlled for and hormonal influences on global-local processing and hemispheric asymmetries have also been discussed. For instance, it has been demonstrated that the global advantage effect is reduced during the luteal phase of the menstrual cycle, when progesterone levels peak (Pletzer et al., 2014). Indeed, higher progesterone levels were directly related to a smaller global advantage effect (Pletzer and Harris, 2018; Pletzer et al., 2014), while higher testosterone levels were related to a larger global advantage effect (Pletzer et al., 2014). Regarding estradiol, no association was observed in the study by Pletzer et al. (2014), while estradiol was not explicitly addressed in Pletzer and Harris (2018). However, several studies point to a role of estradiol for hemispheric lateralization (Hausmann et al., 2013; Weis et al., 2008), as well as brain structural and functional changes along the menstrual cycle (Barth et al., 2016; Pletzer et al., 2018, 2019a,b). Thus, hormonal status has to be controlled for, and all three sex hormones should be considered, when addressing sex differences in global-local processing.

If people with a more local processing style also show more decomposed number processing, a stronger compatibility effect is to be expected in women than in men. This has indeed been demonstrated in multiple studies (Pletzer et al., 2013; Harris et al., 2018; Pletzer et al., 2019a,b), including a large-scale Online study (Nuerk et al., 2001). However, unlike for global-local processing, no study so far has addressed how sex hormones relate to the unit-decade compatibility effect during number comparison or hemispheric asymmetries during number comparison.

Accordingly, the first part of this study, as described in the current manuscript, represents an extension of the study by Harris et al. (2018). On the one hand, we attempt to replicate hemispheric asymmetries and sex differences in the unit-decade compatibility effect, using appropriate eye-fixation control. By demonstrating that major factors influencing basic global-local processing also affect the compatibility effect during number comparison, we seek to further corroborate the evidence that basic visual attentional processes play a role in number processing. Strengthening the link between visual attentional processing and number processing lays the groundwork for studying attentional processes during number comparison in the second part of this study (Schulte et al., n.d.). On the other hand, we address sex hormone influences on the unit-decade compatibility effect and hemispheric asymmetries during number comparison. Due to the similarities between global-local processing and number magnitude processing, we expect the compatibility effect (i) to be smaller when numbers are presented to the left hemifield compared to the right hemifield, (ii) to be smaller in men than in women, (iii) to increase with testosterone levels but decrease with progesterone levels. In addition, we seek to explore whether sex hormones relate to hemispheric asymmetries in number comparison per se or to hemispheric asymmetries in the compatibility effect.

## 2 Methods

### 2.1 Participants

We recruited 87 participants (41 men, 46 women) via social media, e-mail, university courses, flyers, job platforms and word of mouth. Exclusion criteria were left-handedness, alcohol or drug abuse, neurologic, endocrine or psychiatric illness, hormonal contraception or other hormonal medication, age under 18 or over 35 years and glasses. Eight participants (2 men, 6 women) did not complete the study due to technical difficulties.

All female participants had a regular menstrual cycle with a cycle length between 21 and 35 days. Cycle length was averaged over the last three menstrual cycles based on the participant’s self-reported onsets of the last 4 periods. Participant’s used either a mobile App to track their cycle or had the dates noted in a calendar. Variation between individual cycles had to be < 7 days (compare Fehring et al., 2006). Women were tested in the mid-luteal phase of their cycle, i.e. between 3 and 10 days after ovulation. Ovulation was assumed 14 days before the expected onset of next menses as calculated by adding the average cycle duration to the onset of the last menses. However, urinary ovulation kits were not systematically employed, such that anovulatory cycles cannot be strictly excluded. Onset of next menses was controlled via follow-ups. In three women, the onset of next menses was too late or too early. Accordingly, they were excluded from further analyses, which resulted in a final sample of 39 men (mean age 23.35, SD = 3.27) and 37 women (mean age 23.27, SD = 3.59). Age did not differ significantly between women and men (t(74) = 0.11, p = .91, d = 0.03). Furthermore, men and women did not differ in education level (Z = −1.45, p = .11, r = −0.16) and employment status (Z = −0.82, p = .48, r = 0.09), as the majority of participants were students with a general qualification for university entrance and working part time. In addition, IQ was assessed using the Screening version of Raven’s Advanced Progressive Matrices. IQ did not differ significantly between men and women (t_(72)_ = 1.13, p = .26, d = 0.27). Women had a mean cycle duration of 29.15 days (SD = 2.40). The average cycle day of testing was 21.53 (SD = 3.61).

### 2.2 Ethics statement

All participants gave their informed written consent to participate in the study. All data were processed in anonymized de-identified form. All methods conform to the code of ethics constituted by the Declaration of Helsinki (2014).

The institutional guidelines of the University of Salzburg (Statutes of the University of Salzburg^1^) state in §163 (1) that it is necessary to seek ethical approval for research on human subjects if the physical or psychological integrity is affected, the right for privacy or other important rights or interests of the subjects or their dependents are confounded. Paragraph §163 (2) states that it is the decision and responsibility of the PI to decide, whether (1) applies to a study or not. Therefore, no ethical approval for this study was sought out. Non-invasive methods were used on healthy adult volunteers, who willingly gave their informed consent to participate. Accordingly, (1) did not apply. Data were processed in anonymized/de-identified form. Participants were assigned a subject ID (vOOl, v002, etc.), when physically present at the lab, which was used throughout the study.

### 2.3 Number comparison task

**Fig. 1:**
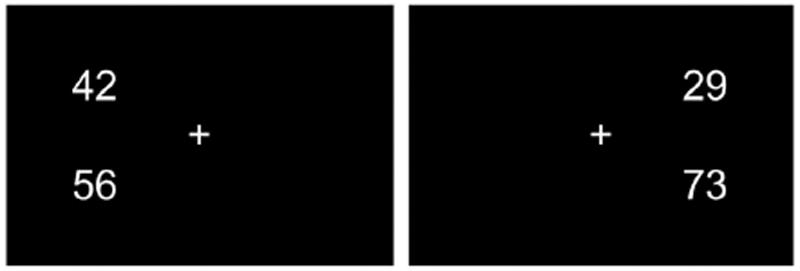
Example stimuli. The left panel shows a compatible item presented to the left hemifield. The right panel shows an incompatible items presented to the right hemifield.

The hemifield number comparison task was one of two tasks participants completed in the framework of this study and consisted of the same 200 items as employed by Harris et al. (2018). The second task is described in Schulte et al. (n.d.). Each item of the hemifield task consisted of two two-digit numbers presented vertically above each other on a computer screen. Participants were seated in a distance of 58 cm from the computer screen. Numbers were 4.44° of visual angle in height and spaced 6.49° apart. Numbers were randomly presented in the left or right hemifield with a distance of 9.78° of visual angle to the fixation cross in the center of the screen (see Fig. 1).

Participants were instructed to press the upper response button on a button box when the upper number was larger and the lower response button when the lower number was larger. In half of the items the upper number was larger, in the other half, the lower number was larger. Among the items presented to the left and right hemifield respectively, 20 comprised so called within-decade (WD) items. In WD items both numbers contain the same decade digit (e.g. 64 < 68), i.e. those items can only be solved by focusing on the unit digits. These items were included as filler items to avoid participants forming a strategy of focusing only on decade digits. Among the remaining 80 items presented to each hemifield, 40 were compatible (higher number has higher unit digit, e.g. 78 vs. 21) and 40 were incompatible (higher number has lower unit digit, e.g. 71 vs. 28). All items consisted of 4 different digits and numbers involved ranged from 21 to 98. Problem size, decade distance, unit distance and parity were matched between stimulus categories (for details, see Harris et al., 2018). At the beginning of each trial, a fixation control was implemented to ensure that participants fixated the center of the screen. Once fixation was confirmed, number comparison trails were presented either to the left or right hemifield for 200 ms. The short presentation time ensures processing in the respective hemispheres as participants do not have the possibility to shift their gaze and look directly at the numbers. Participants had three seconds to decide which number was larger, before the next trial started. Fixations and eye movements were recorded with an EyeLink 1000 eye tracker (SR Research, Ontario, Canada). The eye tracker was calibrated and validated by a 5-point calibration routine. The criterion for successful calibration was an average error of < 0.5° (M = 0.36°, SD = 0.08). Reaction time and accuracy were recorded for each item.

### 2.4 Hormone analysis

Participants gave three saliva samples of 2 ml via the passive drool method throughout the study. One in the beginning, after rinsing their mouth and filling in the informed consent and screening questionnaires. One between tasks and one in the end. Since the experiment lasted about 60 min, about 30 min passed between samples. Samples were frozen at −20°C until hormone analysis. Prior to analysis, saliva samples were centrifuged twice at 3000 rpm for 15 and 10 min, respectively. Saliva from the three samples was pooled prior to analyses to account for fluctuation in hormone release and saliva production. Pooling has the advantage of providing a more stable assessment for the average hormone levels throughout the experiment, but may - in the case of testosterone - have the disadvantage that diurnal changes or pulsatile variations of potential functional relevance are averaged out. DeMediTec salivary ELISA kits were used to determine the levels of testosterone, progesterone and estradiol in each sample. Table 1 shows the average hormone values for men and women. One female progesterone value and one male estradiol value were excluded as outliers since they exceeded the group mean by > 3 standard deviations. Estradiol did not differ significantly between men and women (t_(72)_ = −1.06, p = .29, d = −0.25), which is not unusual given that estradiol levels only reach peak-levels in women during the pre-ovulatory cycle phase (Shultz et al., 2005; Pletzer et al., 2019a,b). Progesterone was significantly lower (t_(72)_ = −6.87, p < .001, d = −1.59) and testosterone significantly higher in men compared to women (t_(73)_ = 10.57, p < .001, d = 2.44). All hormone levels were highly inter-correlated in both men and women (all r > 0.39, all p < .05), with the exception of estradiol and testosterone in men (r = 0.13, p = .44).

### 2.5 Statistical analysis

Statistical analysis was performed via JASP 0.8.1.1. WD items were excluded from analyses and only RT to correctly solved items were included. In a first step, it was evaluated whether the effects of hemi-field and sex on the compatibility effect in number comparison as demonstrated in Harris et al. (2018) could be replicated using adequate fixation control as implemented in the present study. To that end, behavioral data (RT and accuracy) were compared between groups and conditions using repeated measures ANOVAs. 2×2×2 ANOVAs with the within-subjects factors *compatibility* and *hemifield* and the between-subjects factor *sex* were performed to evaluate whether sex and hemifield modulated the compatibility effect in RT and accuracy. In a second step, hormonal influences were assessed. To evaluate the relationship of sex hormones to RT and accuracy, to the compatibility effect, and to hemispheric asymmetries (hemifield effect), they were entered as covariates in the ANOVAs described above. If significant effects of sex hormones were observed in diese ANCOVA-designs, Pearson-correlation coefficients were computed to assess the directionality of the effect.

## 3 Results

### 3.1 Effects of hemifield and sex on the compatibility effect during number comparison

**Fig. 2:**
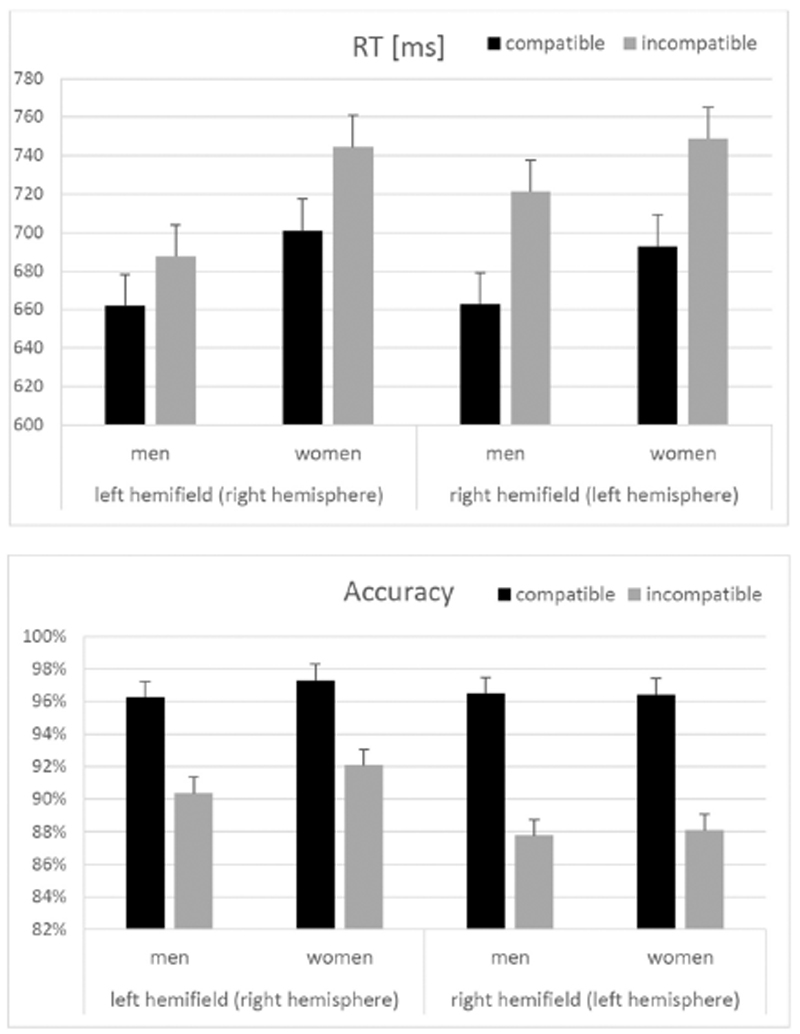
Reaction time (RT) and accuracy for each category in men and women. A compatibility effect was observed in each category. In both RT and accuracy, the compatibility effect was significantly larger in the right hemifield (left hemisphere) than in the left hemifield (right hemisphere). Error bars represent standard errors.

Average RT and accuracy for each category in men and women are illustrated in Fig. 2. We found a significant main effect of compatibility on RT (F_(1,74)_ = 186.84, p < .001, η^2^ = 0.71) and accuracy (F_(1,74)_ = 150.17, p < .001, η^2^ = 0.67). Reaction times were slower and accuracy was lower in incompatible than in compatible items. Hemifield had no significant main effect on RT (F(_1,74_) = 3.72, p = .06, η^2^ = 0.05), but a significant main effect on accuracy (F_(1,74)_ = 8.37, p = .005, η^2^ = 0.10). Accuracy was significantly higher in the left hemifield compared to the right hemifield. Sex had no significant main effect on RT or accuracy and did not interact significantly with each other (all F < 3.58, all p > .06).

Hemifield showed a significant interaction with the compatibility effect in both RT and accuracy (RT: F_(1,74)_ = 17.20, p < .001, η^2^ = 0.18; accuracy: F_(1,74)_ = 9.31, p = .003, η^2^ = 0.11). The compatibility effect was significantly larger in the right hemifield (left hemisphere) than in the left hemifield (right hemisphere).

The compatibility*sex interaction did not reach significance in either RT or accuracy (all F < 1.57, all p > .21). The hemifield*sex interaction was significant in the analysis of RT (F_(1,74)_ = 4.88, p = .03, η^2^ = 0.06), but did not reach significance in the analysis of accuracy (F_(1,74)_ = 0.87, p = .36, η^2^ = 0.01). In men, reactions were faster in the left hemifield (672 ms) compared to the right hemifield (689 ms; t_(38)_ = −3.03, p = .004; d = 0.49), while in women no differences between left (723 ms) and right hemifield (722 ms) were observed (t_(36)_ = 0.19, p = .85, d = 0.03). The threefold interaction between compatibility*hemifield*sex did not reach significance in eitiier, RT or accuracy (both F < 2.45, both p > .12).

### 3.2 Relation of sex hormones to hemispheric asymmetries during number comparison

Sex hormones were not significantly related to accuracy per se and did not interact with any other factors (all F < 3.69, all p > .05). Furthermore, estradiol was not related to overall RT and did not interact with any other factors (all F < 0.83, all p > .37).

**Fig. 3:**
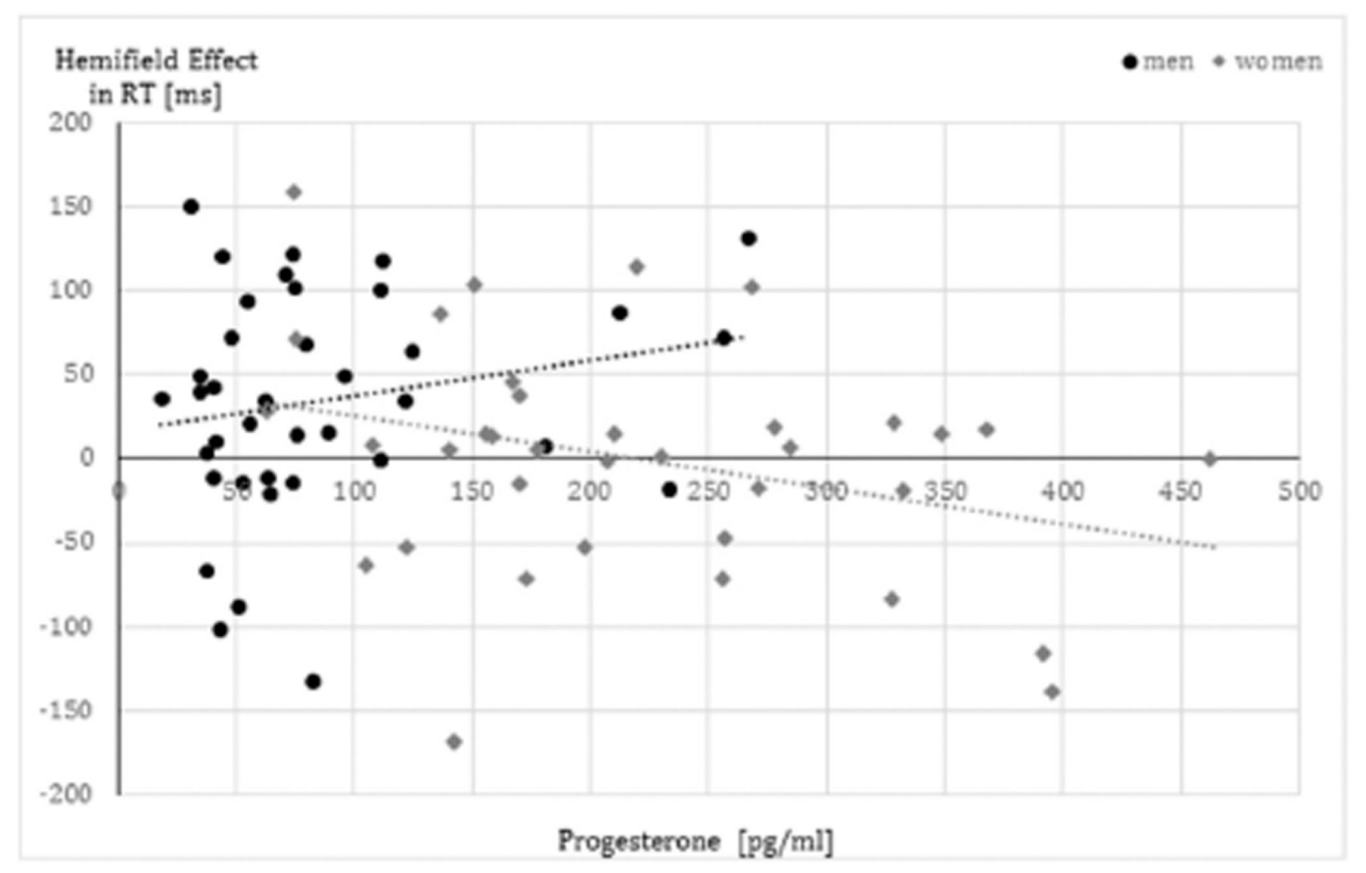
Relationship of progesterone to hemispheric asymmetries in RT in men and women. The hemifield effect was calculated by subtracting reaction times (RT) to stimuli in the right hemifield (left hemisphere) from RT to stimuli in the left hemifield (right hemisphere). Accordingly, positive values indicate faster processing in the left compared to the right hemisphere, while negative values indicate faster processing in the right compared to the left hemisphere. Progesterone relates positively to the hemifield effect in men, but negatively in women. Accordingly in men, progesterone was positively related to faster processing in the left compared to right hemisphere, while in women the opposite relationship was observed.

In the analysis of progesterone for RT we observed a significant hemifield*sex*progesterone interaction (F_(1,70)_ = 4.38, p = .04, η^2^ = 0.06). The main effect of progesterone and all other interactions with progesterone were non-significant (all F < 1.03, all p > .31). In men, the hemifield effect was positively related to progesterone levels (r = 0.21), while in women, the hemifield effect was negatively related to progesterone levels (r = −0.31) – compare Fig. 3. Accordingly, in men, progesterone was positively related to faster processing in the left compared to right hemisphere, while in women the opposite relationship was observed.

**Fig. 4:**
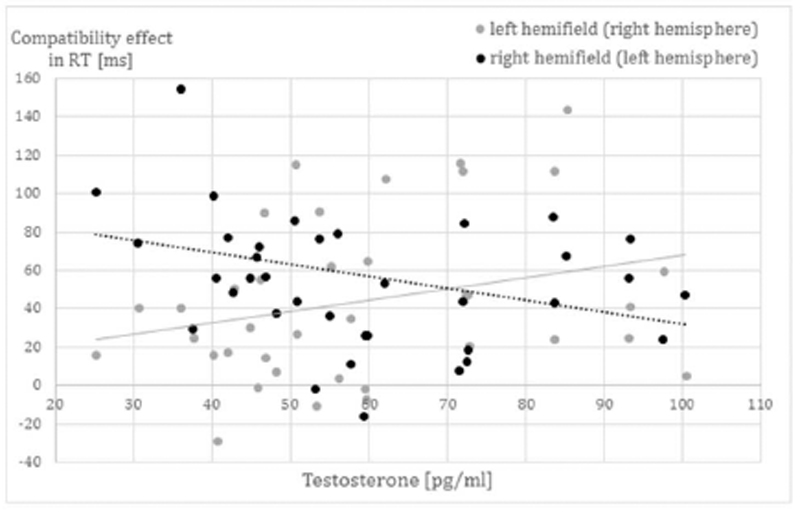
Relationship of testosterone to hemispheric asymmetries in the compatibility effect in RT in women. The compatibility effect was calculated by subtracting the average response times to compatible stimuli from the average response times to incompatible stimuli. Testosterone was related to an increased compatibility effect in the left hemifield, but a decreased compatibility effect in the right hemifield. Accordingly testosterone related to reduced hemispheric asymmetries in the compatibility effect in women. Women with lower testosterone levels had a larger compatibility effect in the right hemifield (left hemisphere).

In the analysis of testosterone for RT, there was a significant 4-fold interaction between testosterone*sex*hemifield*compatibility (F_(1,71)_ = 13.13, p < .001, η^2^ = 0.13). The main effect of testosterone and all other interactions with testosterone were non-significant (all F < 3.78, all p > .05). To resolve this interaction, separate ANCOVAs including the compatibility*hemifield*testosterone interaction were run for men and women. In men, the interaction was non-significant (F_(1,36)_ = 3.33, p = .08, η^2^ = 0.09). In women, the interaction was significant (F(_1,35_) = 8.52, p = .006, η^2^ = 0.20). In women, testosterone was positively related to the compatibility-effect in the left hemifield (r = 0.27), but negatively to the compatibility effect in the right hemifield (r = −0.28) - as illustrated in Fig. 4. Accordingly, testosterone in women relates positively to decomposed number processing in the right hemisphere, but negatively to decomposed number processing in the left hemisphere.

## 4 Discussion

It has recently been demonstrated that the tendency to process multi-digit numbers in a holistic or decomposed manner (as indexed by the unit-decade compatibility effect) relates to global-local visual processing (Pletzer et al., n.d, Pletzer et al., 2019b). The present study set out to investigate, whether factors related to global-local processing also relate to the unit-decade compatibility effect during number comparison. In particular we thought to replicate (i) hemispheric asymmetries and (ii) sex differences in the unit-decade compatibility effect and explore the relationship of sex hormones to (iii) the unit-decade compatibility effect and (iv) hemispheric asymmetries during number comparison.

As expected, we observed a stronger compatibility effect, when numbers were presented to the right hemifield (left hemisphere) compared to the left hemifield (right hemisphere). This replicates previous findings by Harris et al. (2018), who also report hemispheric asymmetries in number comparison. However, this previous study has not used adequate fixation control during the hemifield experiment. It is therefore not surprising, that the effect of hemifield in the present study is much stronger than in the study of Harris et al. (2018). These results support the hypothesis that processing of multi-digit numbers is more decomposed, when it occurs in the left hemisphere and validates the link to global-local processing.

However, unlike previous studies, we did not observe a significant sex difference in the compatibility effect (Pletzer et al., 2013; Harris et al., 2018; Huber et al., 2017; Pletzer et al., 2019a,b). While the compatibility effect in RT was numerically larger in women compared to men, particularly in the left hemifield (compare Fig. 1), this effect did not reach significance. However, previous studies show that, even though sex is the strongest predictor for the compatibility effect (Huber et al., 2017), the effect size is small to moderate (R^2^ = 0.037). It is therefore possible that the present study was underpowered to detect this effect. Also, no sex differences in the compatibility effect were found in the second number comparison task employed in this sample (Schulte et al., n.d.). Furthermore, some studies also report non-significant findings for sex differences in the global advantage effect (Kimchi et al., 2009; Pletzer and Harris, 2018).

Most importantly, this study is the first to also assess the relationship of sex hormones to the compatibility effect and hemispheric asymmetries therein. As previously reported for global-local processing (Pletzer et al., 2014), progesterone and testosterone emerge as hormones of interest, while no relationship to estradiol was observed. However, progesterone and testosterone do not relate to the compatibility effect per se. Rather, progesterone relates to hemispheric asymmetries during number comparison irrespective of compatibility, while testosterone relates to hemispheric asymmetries in the compatibility effect. Both results were dependent on the sex of participants. Progesterone relates positively to hemispheric asymmetries in men, but negatively in women. Testosterone relates negatively to hemispheric asymmetries in the compatibility effect only in women.

It has previously been demonstrated that functional hemispheric asymmetries are related to sex hormones with either estradiol or progesterone relating to reduced lateralization of specific cognitive functions (Hausmann et al., 2000; Hausmann and Güntürkün, 2000; see Hausmann and Bayer, 2010 for a review; see Hausmann et al., 2013 for an EEG study, see Weis et al., 2008 for an fMRI study). This has been attributed to progesterone and/or estradiol strengthening the inter-hemispheric transfer of information by either reducing inter-hemispheric inhibition from the dominant on the non-dominant hemisphere (Hausmann et al., 2000) or increasing inter-hemispheric communication (e.g. Hausmann et al., 2013). The fact that larger levels of female sex hormones relate to more bilateral processing patterns has also been used to explain a general lack of lateralization, i.e. more bilateral processing, in women than in men (McGlone, 1980; Shaywitz et al., 1995; Hausmann and Bayer, 2010; Hausmann and Güntürkün, 2000). In line with these previous findings, we did observe stronger hemispheric asymmetries favoring the right hemisphere in men compared to women. Even though accuracy was higher in the left hemifield (right hemisphere) for all participants, men also react faster when stimuli are presented to the left hemifield (right hemisphere), while no hemifield-related differences in RT were observed in women. Furthermore, in women, the hemifield effect in RT was negatively related to progesterone levels, i.e. hemispheric asymmetries were even smaller in women with higher progesterone levels. This suggests that - in accordance with previous findings - the reduced hemispheric asymmetries in women were indeed related to higher progesterone levels. In men however, the opposite pattern was observed. Their hemifield effect was positively related to progesterone levels, suggesting that hemispheric asymmetries were even larger in men with larger progesterone levels. While this pattern is opposite to what has been previously hypothesized, it has to be pointed out, that previous studies demonstrating a relationship of progesterone to hemispheric asymmetries/lateraliza-tion focused on menstrual cycle related changes in women (Heister et al., 1989; Hampson, 1990; Rode et al., 1995; Hausmann et al., 2000; Hausmann and Güntürkün, 2000; Bayer and Erdmann, 2008; see Hausmann and Bayer, 2010 for a review; see Hausmann et al., 2013 for an EEG study, see Weis et al., 2008 for an fMRI study). Accordingly, the relationship of progesterone to hemispheric asymmetries in men has not been previously addressed. Our result suggests that progesterone plays a different role in inter-hemispheric interactions for men and women. Apparently, it is not so much the fact, that women have higher progesterone levels than men that accounts for sex differences in hemispheric asymmetries. Rather, progesterone even enhances these sex differences, by increasing hemispheric asymmetries in men, but reducing them in women.

A role of testosterone in that respect has not previously been explored. It is generally assumed that testosterone enhances right hemisphere functioning (Toga and Thompson, 2003). Furthermore, due to sex differences in hemispheric lateralization with generally stronger lateralization in men than in women (McGlone, 1980, Shaywitz et al., 1995; Hausmann and Bayer, 2010; Hausmann and Güntürkün, 2000), it can be assumed that testosterone acts as a counterplayer to estradiol and progesterone in that respect. In the present study, however, testosterone related to reduced hemispheric asymmetries in the compatibility effect in women. Thus for women, like progesterone, testosterone relates to more similar behavioral patterns in the left and right hemifield. It is however important to point out, that testosterone was not related to functional hemispheric asymmetries in the task per se, but in an effect that is indicative for a certain processing style during the task. It is possible, that sex hormones play a different role for hemispheric asymmetries in such processing styles, than for overall hemispheric asymmetries in a task. While the latter have been related to hemispheric preferences for certain stimulus materials (e.g. verbal - left; spatial -right, Ocklenburg and Gunturkun, 2012), processing styles trace back to basic visual attention. However, only few studies have assessed the role of sex hormones for hemispheric asymmetries in basic visual attention or processing styles. In a recent neuroimaging study on the relationship of sex hormones to lateralization during global-local processing, we also observed hemispheric asymmetries in the global advantage effect to be the smaller, the higher the participant’s testosterone levels (Pletzer and Harris, 2018). These findings are comparable to results of the present study. In the same study, testosterone was also identified as a modulator variable in the relationship between inter-hemispheric connectivity and lateralization.

In summary the present study confirms hemispheric asymmetries during number comparison, thereby validating the link between number magnitude processing and global-local visual processing. In addition, this study is the first to demonstrate a relation between sex hormones and hemispheric asymmetries during number comparison. Thus, this study adds to the growing evidence that sex hormones relate to hemispheric asymmetries in cognitive functions. Our results demonstrate opposite roles of progesterone for hemispheric asymmetries in men and women and suggest an important role of testosterone, which has so far hardly been investigated.

## Figures and Tables

**Table 1:** Hormone values (mean ± SD) for men and women.

	Men	Women
Estradiol (pg/ml)	4.06 ± 1.13	4.48 ±2.14
Progesterone (pg/ml)	86.65 ± 63.38***	220.17 ± 100.72
Testosterone (pg/ml)	120.82 ± 29.79***	**59.19 ± 19.51**

*** Significantly different from women (p < .001).

## Footnotes

1
https://www.ncbi.nlm.nih.gOv/pmc/articles/PMC5355435/#fn01.
